# Evolution of mammalian sensorimotor cortex: thalamic projections to parietal cortical areas in *Monodelphis domestica*

**DOI:** 10.3389/fnana.2014.00163

**Published:** 2015-01-07

**Authors:** James C. Dooley, João G. Franca, Adele M. H. Seelke, Dylan F. Cooke, Leah A. Krubitzer

**Affiliations:** ^1^Center for Neuroscience, University of California, DavisDavis, CA, USA; ^2^Instituto de Biofísica Carlos Chagas Filho, Universidade Federal do Rio de JaneiroRio de Janeiro, Brazil; ^3^Department of Psychology, University of California, DavisDavis, CA, USA

**Keywords:** marsupial, cortical evolution, multimodal cortex, 3a, S1, posterior parietal, thalamocortical projections, comparative neuroanatomy

## Abstract

The current experiments build upon previous studies designed to reveal the network of parietal cortical areas present in the common mammalian ancestor. Understanding this ancestral network is essential for highlighting the basic somatosensory circuitry present in all mammals, and how this basic plan was modified to generate species specific behaviors. Our animal model, the short-tailed opossum (*Monodelphis domestica*), is a South American marsupial that has been proposed to have a similar ecological niche and morphology to the earliest common mammalian ancestor. In this investigation, we injected retrograde neuroanatomical tracers into the face and body representations of primary somatosensory cortex (S1), the rostral and caudal somatosensory fields (SR and SC), as well as a multimodal region (MM). Projections from different architectonically defined thalamic nuclei were then quantified. Our results provide further evidence to support the hypothesized basic mammalian plan of thalamic projections to S1, with the lateral and medial ventral posterior thalamic nuclei (VPl and VPm) projecting to S1 body and S1 face, respectively. Additional strong projections are from the medial division of posterior nucleus (Pom). SR receives projections from several midline nuclei, including the medial dorsal, ventral medial nucleus, and Pom. SC and MM show similar patterns of connectivity, with projections from the ventral anterior and ventral lateral nuclei, VPm and VPl, and the entire posterior nucleus (medial and lateral). Notably, MM is distinguished from SC by relatively dense projections from the dorsal division of the lateral geniculate nucleus and pulvinar. We discuss the finding that S1 of the short-tailed opossum has a similar pattern of projections as other marsupials and mammals, but also some distinct projections not present in other mammals. Further we provide additional support for a primitive posterior parietal cortex which receives input from multiple modalities.

## Introduction

The emergence of a six-layered neocortex and its diversification from 10 to 12 areas to hundreds of cortical areas is the hallmark of mammalian evolution (Krubitzer, 2007; Kaas, 2011). Our laboratory is interested in how the shared features of all mammalian brains inform the likely characteristics of the ancestral mammalian brain, how phenotypic transformations such as additional cortical areas arise, and the constraints imposed on evolving and developing brains (Krubitzer and Dooley, 2013). There are a number of cortical areas that are present in all mammalian species investigated, including primary sensory areas, second sensory areas, and a few additional cortical areas not exclusively related to unimodal sensory processing (for review, see Kaas, 2011).

Interestingly, the organization and number of motor areas in the cortex is quite different in different mammals, and whether the common ancestor of all mammals actually possessed even a primary motor area (M1) is unclear (Kaas, 2011). While a separate motor area, rostral to the primary somatosensory area (S1) and containing a complete and mirrored body representation is a feature shared among eutherian mammals, this group represents just one of three major clades of mammals. Further, even in placental mammals the relationship between M1 and S1 is variable (e.g., Donoghue et al., 1979; Donoghue and Wise, 1982). While only three extant species of Monotremes are available for study, the diversity and relative availability of different marsupial species has revealed motor representations that are surprisingly divergent from those seen in placental mammals (Karlen and Krubitzer, 2007). Among marsupials, American opossums (order Didelphimorphia) are both the largest order (with over 100 species) and form the earliest radiation of marsupials (Kemp, 2005), which occurred approximately 150 million years ago (Nilsson et al., 2010). Within this order is a small South American marsupial, the short-tailed opossum (*Monodelphis domestica*). Short-tailed opossums appear to share many characteristics with the ancestral mammal, including skull morphology, body size, brain size and the ecological niche that they occupy (Rowe et al., 2011), suggesting that these animals could serve as good models for the brain organization of our earliest ancestors.

It is not surprising then, that among marsupials studied, opossums are the only order which have been consistently and convincingly demonstrated to lack a separate motor cortex in which microstimulation evokes movements (Magalhaes-Castro and Saraiva, 1971; Beck et al., 1996; Frost et al., 2000); for review see (Karlen and Krubitzer, 2007). In all opossum species in which microstimulation techniques have been used, movements could only be initiated by stimulating S1. Specifically, in both the big-eared opossum (*Didelphis aurita*) and the Virginia opossum (*Didelphis virginiana*), movements could be elicited when stimulating separate locations throughout the entire extent of S1 (Magalhaes-Castro and Saraiva, 1971; Beck et al., 1996), while in the short-tailed opossum (*Monodelphis domestica*), movements could only be elicited when stimulating the face representation of S1 (Frost et al., 2000). This partial motor map (face but not body representation) of the short-tailed opossum is in direct contrast to the results observed in the big-eared opossum and the Virginia opossum. While thalamic connectivity of S1 has been investigated previously in Virginia opossums, adding further support for the existence of an entirely overlapping sensorimotor amalgam (Killackey and Ebner, 1973; Donoghue and Ebner, 1981a,b; Foster et al., 1981); to date there has been no study of the thalamic projections to S1 and the surrounding areas in the short-tailed opossum. Thus, the primary aim of the current investigation is to characterize the thalamic projections to numerous somatosensory and surrounding cortical areas of the short-tailed opossum neocortex.

In previous studies, marsupials (including the short-tailed opossum) have been shown to have at least two complete somatotopic representations within the neocortex: S1 and the second somatosensory area, S2 (Beck et al., 1996; Huffman et al., 1999b; Catania et al., 2000; Frost et al., 2000; for review see Karlen and Krubitzer, 2007). Further analysis of electrophysiological recordings, architecture, and connectional patterns by our own and other laboratories has supported the presence of additional fields associated with somatosensory processing, including a rostral field (SR) as well as a caudal field (SC; Beck et al., 1996; Elston and Manger, 1999; Huffman et al., 1999b; Wong and Kaas, 2009; Anomal et al., 2011; Dooley et al., 2013). A third complete somatotopic representation (the parietal ventral area, PV) has been identified in numerous marsupials investigated (Beck et al., 1996; Elston and Manger, 1999; Huffman et al., 1999b), however despite careful exploration, a separate, third somatotopic representation has not been identified in the short-tailed opossum (Catania et al., 2000; Frost et al., 2000). However, the region which has been identified shows characteristics of both S2 and PV, thus we have elected to refer to this area as S2/PV throughout the text. Further, a previous study conducted by our laboratory in the short-tailed opossum identified a multimodal region (MM), caudal to SC and rostral to V2, which had connections with both somatosensory and visual areas of the neocortex (Dooley et al., 2013).

In the present investigation we examined and quantified the thalamic projections to three somatosensory fields in the short-tailed opossum (SR, S1 and SC), as well as the multimodal region (MM). Further, in order to investigate whether different body part representations within S1 were associated with the motor system, thalamic projections restricted to the body or the face representation of S1 were investigated separately and in some cases directly compared. To determine patterns of thalamocortical connectivity, several retrograde anatomical tracers were injected into architectonically and/or electrophysiologically defined locations within these fields and were related to the patterns of projections from architectonically defined thalamic nuclei.

## Methods

Neuroanatomical tracer injections were combined with architectonic analysis in eight adult short-tailed opossums (4 males, 4 females, 76–136 grams) to determine the thalamocortical connections of parietal cortical areas, including both the lateral and medial portions of primary somatosensory cortex (S1, for abbreviations see Table 1), the rostral and caudal somatosensory fields (SR and SC respectively), and the cortical region just caudal to SC (multimodal cortex, or MM). In several cases, electrophysiological recordings were performed to identify the receptive fields for neurons at or surrounding the injection site (for table of injection sites and cases see Table 2). All animals were housed in standard laboratory conditions, with food and water available *ad libitum*. Animals were maintained on either a 12-h light/dark cycle or a 14/10 h light/dark cycle. All protocols were approved by the Institutional Animal Care and Use Committee of the University of California, Davis, and all experiments were performed under the National Institutes of Health's guidelines for the care of animals in research.

**Table 1:** **List of abbreviations used throughout the text**.

**CORTICAL FIELDS**	
3a	Somatosensory area (deep)
A1	Primary auditory cortex
EC	Entorhinal cortex
FM	Frontal myelinated area
M1	Primary motor cortex
MM	Multimodal cortex
PP/PPC	Posterior parietal cortex
S1	Primary somatosensory area
S1 face	Primary somatosensory area, face division
S1 body	Primary somatosensory area, body division
S2	Second somatosensory area
SC	Somatosensory caudal area
SR	Somatosensory rostral area
V1	Primary visual area
V2	Second visual area
**SUBCORTICAL STRUCTURES**	
APT	Anterior pretectum
CeM	Central medial nucleus
CL	Central lateral nucleus
CP	Cerebral peduncle
eml	External medullary lamina
Hb	Habenula
iml	Internal medullary lamina
LGNd	Lateral geniculate nucleus, dorsal division
LGNv	Lateral geniculate nucleus, ventral division
MD	Medial dorsal nucleus
MGNd	Medial geniculate nucleus, dorsal division
MGNm	Medial geniculate nucleus, magnocellular division
MGNv	Medial geniculate nucleus, ventral division
mt	Mammillothalamic tract
MV	Medioventral nucleus
ot	Optic Tract
PAG	Periaqueductal gray
pc	Posterior commissure
Pol	Posterior nucleus (lateral)
Pom	Posterior nucleus (medial)
Pul	Pulvinar
PV	Paraventricular nucleus
Rt	Reticular nucleus
ST	Subthalamic nucleus
VA	Ventral anterior nucleus
VB	Ventral basal nucleus
VL	Ventrolateral complex
VM	Ventromedial nucleus
VMb	Ventral medial basal nucleus
VP	Ventral posterior nucleus
VPl	Ventral posterolateral nucleus
VPm	Ventral posteromedial nucleus
**OTHER ABBREVIATIONS**	
CO	Cytochrome oxidase
CTB	Cholera toxin subunit-B
FE	Fluoro-emerald
fl	forelimb
FR	Fluoro-ruby
vGluT2	Vesicular glutamate transporter 2

**Table 2:** **Asterisks next to case numbers indicate cases for which corticocortical connections were reported previously (Dooley et al., 2013)**.

**Case**	**Area injected + halo**	**Hemisphere injected**	**Tracer**	**Volume injected (μl)**	**Injection area (mm^2^)**	**Injection RF**	**Figure number**
13-114	S1 body	Left	FR	0.2	0.07		6
12-13*	S1 body	Right	FR	0.3	0.11		7
13-126	S1 face	Left	FR	0.2	0.07	Face/Vib	2, 8
09-18*	S1 face	Left	FR	0.3	0.07		
12-18*	S1	Right	FR	0.3	0.06	Vib/Forepaw	9
13-73	S1	Right	FE	0.3	0.07		
08-29*	SC	Left	FE	0.3	0.08		10
09-18*	SC	Left	FE	0.3	0.14		
13-73	SC	Right	FR	0.2	0.12		11
08-80*	MM	Left	FE	0.3	0.07	Unresponsive	12
08-80*	SR	Left	FR	0.3	0.07	Lips	13
13-126	S1 body, SR	Left	CTB	0.15	1.03	Forepaw	2

*Receptive field of injection site is shown, when available*.

### Neuroanatomical tracer injections

Animals were placed in an induction chamber and anesthetized with isoflurane (1–5% per liter O_2_). Following induction, the animal's head was shaved and a specially fitted mask was placed over the animal's nose. Throughout the procedure, the surgical plane of anesthesia was maintained with 1–3% isoflurane. A 2% lidocaine solution was injected subcutaneously at the midline of the scalp and around the ears, and animals were paced in a stereotaxic apparatus. Animals were given dexamethasone (0.4–2.0 mg/kg, IM) and atropine (0.04 mg/kg, IM) at the start of the surgery. Respiration and body temperature were monitored throughout the procedure.

Under standard sterile conditions, an incision was made at the midline of the scalp, the temporal muscle was unilaterally retracted, and a small craniotomy was performed over parietal cortex. In some cases, a photograph was taken over the exposed cortex to record the position of the tracer injections relative to blood vessels. In several cases, electrophysiological recordings were performed to confirm the placement of the injections (Table 2). A small hole was made in the dura over the area of the injection site, and either a custom-beveled Hamilton syringe (Hamilton Co., Reno, NV) or a Hamilton syringe fitted with a glass pipette beveled to a fine tip was lowered ~300–400 μm into the cortex. Between 0.15 and 0.3 μL of 10% Fluoro-emerald (FE, Invitrogen, Carlsbad, CA), Fluoro-ruby (FR, Invitrogen, Carlsbad, CA), or cholera toxin subunit-B (CTB; Sigma-Aldrich, St. Louis, MO) was pressure-injected into the cortex. A total of 12 tracer injections were made in 8 animals with several animals receiving multiple injections (Table 2). Following the injection, the surface of the brain was flushed with sterile saline to remove any remaining tracer on the cortical surface, the craniotomy was covered with either bone wax or an acrylic skull cap (depending on the size of the opening), and the temporal muscle and skin were sutured. Antibiotics (Baytril, 5 mg/kg, IM) and analgesics (buprenorphine, 0.03 mg/kg, IM) were given. Animals recovered for 5–7 days to allow for the transport of the tracer, after which they were euthanized with an overdose of sodium pentobarbital (Beuthanasia; 250 mg/kg, IP), transcardially perfused with 0.9% saline, followed by 2–4% paraformaldehyde in phosphate buffer (pH 7.4), and finally 2–4% paraformaldehyde in 10% phosphate-buffered sucrose. These procedures have been described previously (Dooley et al., 2013).

### Electrophysiological recordings

**Figure 1:**
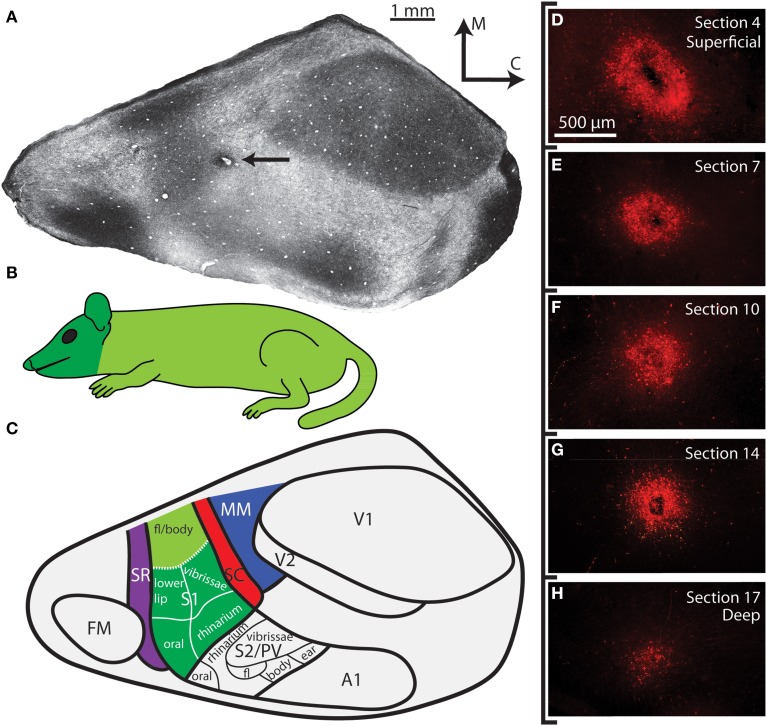
**Cortical organization in *Monodelphis domestica***. **(A)** A tangential section of cortex that has been stained for myelin. The medial wall, caudal wall and piriform cortex have been removed. The arrow points to an injection located in SC. **(B)** An illustration of the body of the short-tailed opossum colored according to the head representation (dark green) and the body representation (light green). **(C)** Schematic of cortical fields and body representations in a brain oriented as in A. Different cortical fields are depicted in different colors; these colors are used in later figures showing the anatomical connections (labeled cell bodies in the thalamus) to indicate the cortical field location of the injection site. Thick lines delineate borders between different cortical areas, while thin lines separate different body part representations within S1 and S2/PV determined in previous electrophysiological recording studies. Boundaries of cortical areas are based on architectural, connectional, and electrophysiological data, while topographic boundaries within S1 are based on electrophysiological experiments. The functional organization of S1 and S2/PV is adapted from Catania et al. (2000). **(D–H)** Superficial to deep sections of the cortical injection in A. Other than being slightly larger in the most superficial section **(D)**, the injection halo remains similar in size through intermediate sections **(E–G)**, before it finally begins to shrink in the final section **(H)**. As with all other cases included in this study, the injection halo included all cortical layers but did not extend into the underlying white matter. See Table 1 for abbreviations.

In three cases (08-80, 09-32, and 12-18) electrophysiological recording experiments were performed following 5–7 days of recovery. When possible, these experiments helped confirm the S1/S2 boundary, the boundary of the face/body representations within S1, as well as the rostral and caudal extent of S1 (Figures 1B,C). For terminal electrophysiological recording experiments, two animals were anesthetized with 1–3% isoflurane and one was anesthetized using 30% urethane in propylene glycol (1.25 g/kg, IP). No differences were noted between maps using different anesthesia methods. Surgical procedures were the same as those described previously, except a larger craniotomy was performed over the entire neocortex and the dura was retracted. Digital images were taken so that electrophysiological recording sites could be related to injection sites and cortical vasculature.

Multiunit electrophysiological recordings of parietal cortex were performed using tungsten microelectrodes (0.010 inches, 0.5–5 MΩ; A-M Systems, Sequim, WA). The electrode was lowered ~400 μm below the pial surface, in layer IV of cortex. Multiunit activity was amplified and filtered (100–5,000 Hz; A-M Systems Model 1800 Microelectrode AC Amplifier; A-M Systems, Carlsborg, WA), monitored through a loudspeaker, and viewed on a computer monitor. At each recording site neural responses to somatosensory stimulation (consisting of light taps, displacement of vibrissae, brushing of skin, hard taps and manipulation of muscles and joints) were recorded and receptive fields were both drawn on illustrations of the body and documented in surgical notes. These methods have been previously described (Dooley et al., 2013). Following electrophysiological recording, animals were euthanized and perfused as described above.

### Histology

Once perfused, the brain was extracted, weighed, and photographed and the cortex was separated from the subcortical structures. In some cases, the hippocampus and basal ganglia were dissected from the cortical hemispheres. All dissected cortices were then manually flattened between glass slides, briefly post-fixed in 4% paraformaldehyde in 10% phosphate-buffered sucrose, then allowed to soak for 12–36 h in 30% phosphate-buffered sucrose. The flattened cortex was then sectioned at 30 μm using a freezing microtome. Alternating cortical sections were stained for myelin (Gallyas, 1979) or mounted immediately for fluorescent microscopy. In cases in which CTB was injected, cortical sections were divided into three series, one of which was reacted for CTB.

Subcortical structures were post-fixed as described above and then allowed to soak in 30% phosphate-buffered sucrose until they sunk in solution (24–48 h). Following this, they were sectioned coronally at 30–40 μm. Tissue was divided into 3 or 4 series. One series in all cases was stained for cytochrome oxidase (CO; Wong-Riley, 1979) while a second was mounted immediately for fluorescent microscopy. When applicable, immunohistochemistry was performed for CTB. Additionally, all cases were stained for either Nissl or processed for vGluT2 expression (mouse monoclonal anti-vGluT2 from Millipore, Billerica, MA; 1:5000).

### Data analysis

**Figure 2:**
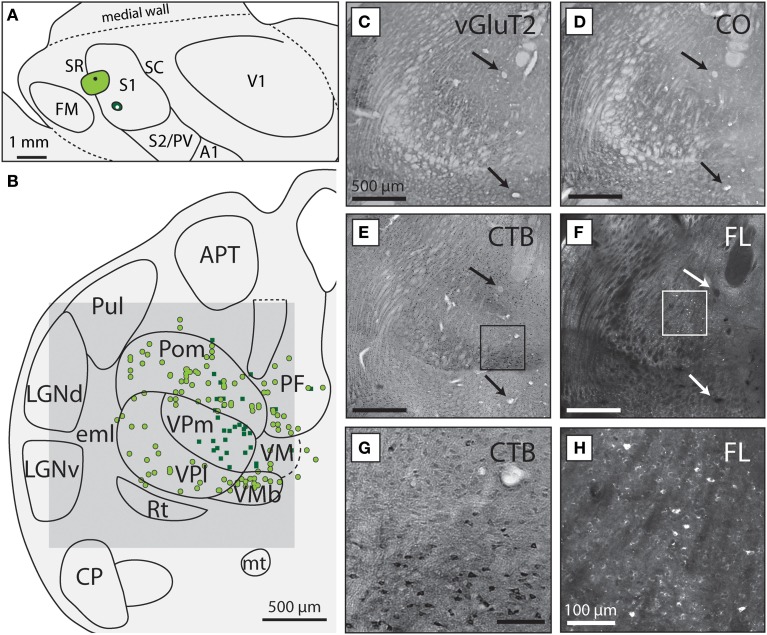
**VPm and VPl project to different body representations within S1. (A)** Reconstruction of the flattened cortical hemisphere from case 13-126 in which one injection of CTB (light green circle) was placed in the S1 body representation and one injection of FR was placed in the S1 face representation (dark green circle). **(B)** Reconstructed thalamic section showing the boundaries of different subcortical nuclei drawn from histologically processed tissue. Dorsal is up, lateral is to the left. Colored dots and squares correspond to labeled neuronal nuclei resulting from each injection, with light green circles projecting to the light green S1 body injection and the dark green squares projecting to the S1 face injection in **(A)**. Dashed lines indicate borders that were more difficult to determine from the sections available. The gray box corresponds to the region of the light field and fluorescent images in **(C–F)**. Light field image of tissue stained for **(C)** vGluT2, **(D)** CO, **(E)** CTB, and **(F)** unstained tissue visualized under fluorescence microscopy. The arrows in each box indicate the presence of blood vessels use to align tissue. The scale bar for **(C–F)** are all 500 μm. The boxes in **(E)** and **(F)** are magnified in **(G)** and **(H)**, respectively, showing **(G)** neurons labeled with CTB and **(H)** FR. The scale bar for **(G,H)** is 100 μm. See Table 1 for abbreviations.

Injections sites and cortical field boundaries were reconstructed as described previously (Campi et al., 2010; Dooley et al., 2013). Briefly, reconstructions of anatomical boundaries from photographs of individual myelin sections were made (Figure 1A), and the boundaries from the entire series were examined and combined into a single reconstruction (Figure 2A; for details, see Seelke et al., 2012). Each reconstructed section contained an outline of the section, blood vessels, tissue artifacts, probes, and architectonic borders of cortical fields. These landmarks were then directly related to sections mounted for fluorescence or processed for CTB, and a comprehensive reconstruction of the neocortex was generated that contained the injection site, injection halo, labeled cells and architectonic boundaries. Corticocortical connections for these cases have been described previously (Dooley et al., 2013). Several injections were found to have spread into surrounding cortical areas, and thus these cases were not included in quantitative analysis and Table 2; however the data were qualitatively described and illustrated as figures (e.g., Figure 2). In all cases included in the study, cortical injections spanned all cortical layers and did not extend into the underlying white matter (Figures 1D–H).

For thalamic sections, labeled cells were plotted using an X/Y stage encoding system (MD Plot, Minnesota Datametrics, St. Paul, MN) mounted to a fluorescent microscope and connected to a computer. Additionally, the tissue outline, blood vessels, and artifacts were plotted, which allowed these sections to be aligned to the anatomical boundaries determined from the Nissl, CO or vGluT2 stained tissue (Figures 2C,D). The location of labeled cells in the plotted sections was combined with adjacent sections to form a single comprehensive reconstruction (Figure 2B). When necessary, brightness and contrast were adjusted for the digital images using Photoshop CS5 (Adobe, San Jose, CA). Additionally, in several instances, multiple photographs of a single cortical or thalamic section were turned into a single composite image using Microsoft Image Composite Editor (Microsoft, Redmond, WA).

Thalamocortical connections were quantified by summing the total number of retrogradely labeled cells in the thalamus and calculating the percentage of labeled cells in a given thalamic nucleus. This allowed us to normalize the data for injections of different sizes. This quantification allowed us to determine connection strength for each thalamic nucleus as follows: Strong: > 10%; Moderate: 9% to 3%; Weak: 3% to 1%; Intermittent: < 3% and not present in all cases.

## Results

The goal of these studies was to determine the thalamocortical connections of parietal somatosensory areas as well as the multimodal region in the short-tailed opossum; with the ultimate goal of comparing these connections with other marsupials as well as with eutherian mammals. In the following results we first describe how cortical field boundaries were determined and the extent to which our injection sites were restricted to the different areas of interest. Next, we define the different nuclei in the thalamus by their appearance in histologically processed tissue. Finally, we describe the patterns of thalamocortical connections of S1, SC, SR, and MM and quantify the density of projections from different thalamic nuclei.

### Determination of cortical field boundaries

A subset of the cases used in this study were also used in our previous study of corticocortical connections of parietal cortex in *Monodelphis domestica* (Table 2; Dooley et al., 2013). In addition to our previous publication, more extensive descriptions of the organization of different cortical fields in this species can be found in other studies by our own and other laboratories (Huffman et al., 1999b; Catania et al., 2000; Frost et al., 2000; Kahn et al., 2000a; Karlen et al., 2006; Karlen and Krubitzer, 2009; Wong and Kaas, 2009). In many of these studies, myelin stains were directly compared to electrophysiologically identified boundaries (Catania et al., 2000). Additionally, the neocortex of short-tailed opossums has been assessed in sagittal and coronal sections, demonstrating the laminar distribution of myelin and CO (Wong and Kaas, 2009). As in the present study, the primary sensory areas of the short-tailed opossum, including S1, V1, and A1, were densely myelinated and their borders could be readily determined (Figure 1A, see Table 1 for abbreviations). SR and SC were lightly myelinated strips of cortex approximately 0.5 mm wide and were located directly rostral and caudal to S1 respectively (Figure 1). V2 was identified as a lightly myelinated strip approximately 0.5 mm at the rostral boundary of V1. Multimodal cortex (MM) was a very lightly myelinated region caudal to SC and rostral to V2. S1 contains a representation of the entire contralateral body surface, with the hindpaw represented most medially and the rhinarium and oral structures represented most laterally (Figures 1B,C; Catania et al., 2000). Thus, S1 can be further divided functionally into body (S1 body) and face (S1 face) divisions, generally separated by a small band of lightly myelinated cortex. These techniques were utilized in S1 injections in the present study in order to determine the location of our injection sites relative to the body or face representations.

### Architectonic subdivisions of thalamus

Subdivisions of the thalamus were delineated using sections stained for CO and either Nissl substance or vGluT2, and many of the nuclei described here have been previously distinguished by our own and other laboratories (see Turlejski et al., 1994; Huffman et al., 1999b; Karlen et al., 2006; Olkowicz et al., 2008). The boundaries of thalamic nuclei in short-tailed opossums were also compared to previously published thalamic boundaries reported in the closely related Virginia opossum (Oswaldo-Cruz and Rocha-Miranda, 1968; Jones, 2007).

**Figure 3:**
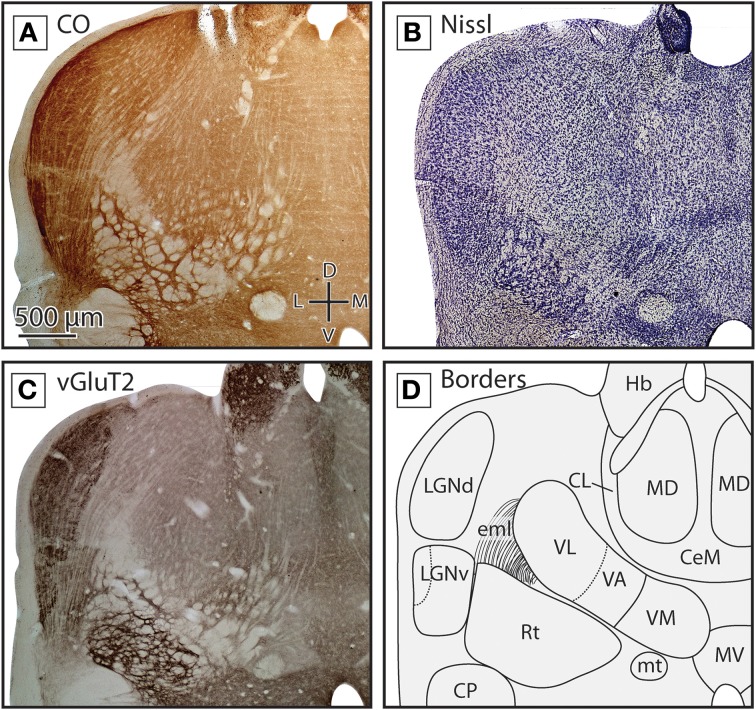
**Boundaries of nuclei in rostral sections of the dorsal thalamus in the short-tailed opossum**. Three different stains were used to delineate the boundaries of thalamic nuclei including CO **(A)** Nissl **(B)** and vGluT2 **(C)**. Reconstructions of thalamic nuclei derived from these stains are drawn in **(D)**. Midline nuclei, including CeM, CL and MD, can be observed using each stain, but are most obvious in Nissl and vGluT2. Ventral and lateral to midline nuclei, VL, VA, and VM can also be seen. VA and VM boundaries are most apparent in vGluT2 **(C)**. Lateral to these structures, LGNd and LGNv can be identified. The boundaries of these structures can be readily identified in CO **(A)** and vGluT2 **(C)** stained tissue. In all images, dorsal is up and lateral is to the left. Scale bar in **(A)** is 500 μm, and is the same for all images.

In the rostral-most sections where label was found, numerous midline nuclei were identified. CeM was located at the midline, was darkly stained, and had densely packed neurons. It was most apparent in Nissl-stained tissue, but was also visible in CO and vGluT2 preparations (Figures 3A–D). Dorsal and lateral to CeM lies another darkly stained nucleus termed CL (which is part of the intralaminar group; Figures 3A–D). As with CeM, CL is most apparent in Nissl-stained tissue but can also be seen in tissue stained for CO and vGluT2. Medial to CL and dorsal to CeM is a large oval nucleus, MD, that has moderate neuronal packing density in Nissl-stained sections (Figure 3B), and stains lightly for both CO and vGluT2 (Figures 3A,C). Boundaries separating these nuclei can be readily determined by the sharp contrast between the darkly stained CeM and CL and the lightly stained MD.

**Figure 4:**
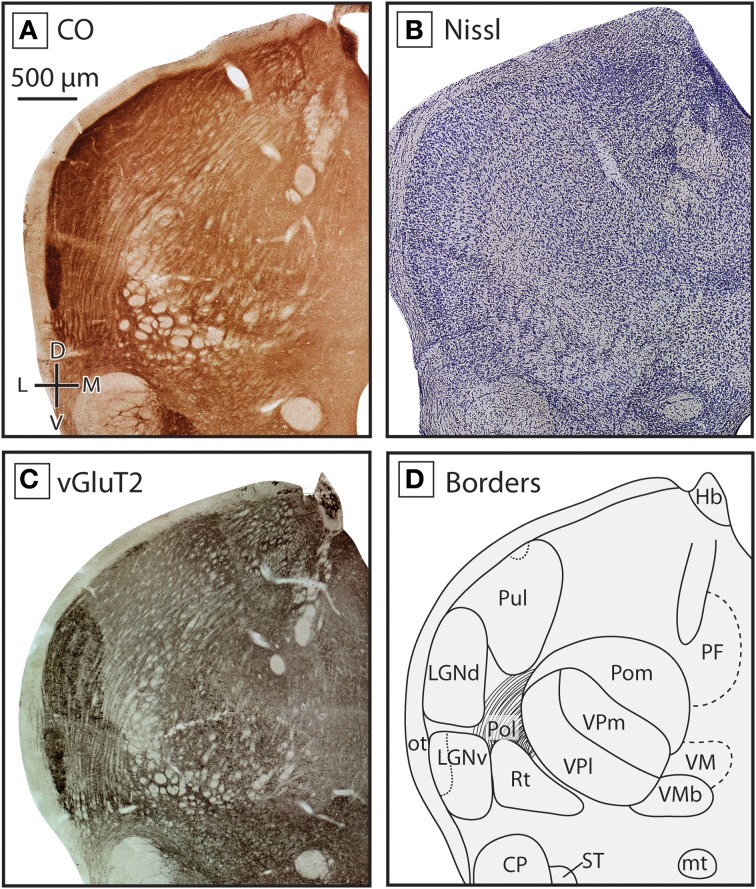
**Boundaries of nuclei in the dorsal thalamus at the level of the ventral posterior nucleus in the short-tailed opossum**. As in the previous figure, three different stains were used to delineate boundaries; CO **(A)**, Nissl **(B)**, and vGluT2 **(C)**, with reconstructions derived from these stains illustrated in **(D)**. The ventral posterior complex, including VPm and VPl can be seen as a darkly staining region in CO **(A)** and vGluT2 **(C)** stained tissue, with VPm staining more darkly. VMb can be seen medial to VP as a lightly stained line of tissue between two darkly stained sections. Lateral to VP, eml stains lightly in all three series. At this level of the thalamus, Pol is intermingled with eml. As in Figure 3, LGNd and LGNv are lateral to the ventral posterior complex, and are most apparent in CO **(A)** and vGluT2 **(C)** stained tissue. Near the midline, PF can be seen in CO **(A)** and vGluT2 **(C)** stained tissue, being slightly darker than the surrounding nuclei. Finally, dorsal to VMb and medial to VP, the caudal extent of VM could be identified as a lightly stained nuclei in all three series. Conventions as in Figure 3.

Lateral to these midline nuclei in more rostral portions of the thalamus we observed labeled neurons in the ventral anterior and the ventrolateral nuclei (VA and VL, respectively). The boundaries between these two nuclei were not always distinct in CO and Nissl-stained tissue, and so throughout the quantitative analysis performed in these studies they were combined into a single category (“VL/VA”). Both of these nuclei stained moderately for CO and showed a medium neuronal packing density in Nissl stains (Figures 3A,B). When tissue was stained for vGluT2, VA stained more darkly than VL and was located medial and ventral to VL (Figure 3C, boundary denoted by the dotted line in Figure 3D). Just medial to VA was VM, a densely packed nucleus that stained darker than the surrounding nuclei in Nissl-stained sections (Figure 3B). However, VM stained moderately for CO and lightly for vGluT2 (Figures 3A,C). VM extended further caudal than VL and VA (Figures 4A–D).

The reticular nucleus, Rt, could be identified throughout most of the rostrocaudal extent of the thalamus (e.g., Figures 3, 4). Rt was heterogeneous in appearance due to the number of fiber bundles passing throughout this nucleus. Despite its heterogeneous appearance, Rt stained darker then the surrounding tissue in all stains, and appeared particularly dark in tissue stained for vGluT2 (Figures 3C, 4C). Throughout most of its rostral-caudal extent, Rt was bounded dorsomedially by the external medullary lamina (eml), which was clearly visible in both CO and vGluT2 stained tissue as a very lightly stained structure (see Figures 3A,C, 4A,C).

The ventral posterior nucleus (VP) was located caudal to VL and VA. It is bordered ventrally by iml and Rt and laterally by eml. VP stained darkly for CO, Nissl and vGluT2 (Figures 4A–C). VP could be further subdivided into lateral (VPl) and medial (VPm) divisions, with VPl corresponding to the representation of the body and VPm corresponding to the representation of the face. Further, VPm has been described as having a higher density of cells compared to VPl, and thus appeared darker than surrounding tissue in a Nissl stain, and stained darker for CO. The boundary between VPl and VPm was most apparent in tissue stained for CO (see Figure 4A). Dorsal, caudal, and in some sections lateral to VP was the posterior nucleus composed of both a medial (Pom) and lateral (Pol) division. Pom extended rostrally and is located just medial to VP, staining lightly for both CO and Nissl (Figures 4A,B). Medial to VP we identified the ventral medial basal nucleus (VMb). This nucleus stained moderately for CO and Nissl, and often appeared lighter than regions immediately dorsal, ventral and medial to it (Figures 4A,B). In tissue stained for vGluT2, VMb stained darkly (Figure 3C).

As has been described previously (Huffman et al., 1999a; Karlen et al., 2006), in *Monodelphis* both LGNv and LGNd stained darkly for CO, and are separated from each other by a thin lightly stained region (see Figures 3A, 4A). LGNv contained a lateral portion that stained very darkly for both CO and in Nissl. LGNv extended medially to the lateral border of Rt (Figures 3A–D, 4A–D). In tissue stained for vGluT2, LGNd stained much more darkly than the surrounding tissue, and individual laminae could be observed (Figures 3C, 4C). Dorsomedial to the LGNd is the pulvinar (Pul, previously termed the lateral posterior thalamic nuclei; Kahn et al., 2000b). Neurons in Pul showed a moderate packing density in tissue stained for Nissl and CO (Figures 4A,B). Pul was moderately stained by vGluT2, and the caudal subdivision stained more intensely near the dorsal-most extent (Figure 4C; Baldwin et al., 2013). This subdivision is indicated with a dotted line in Figure 4D.

**Figure 5:**
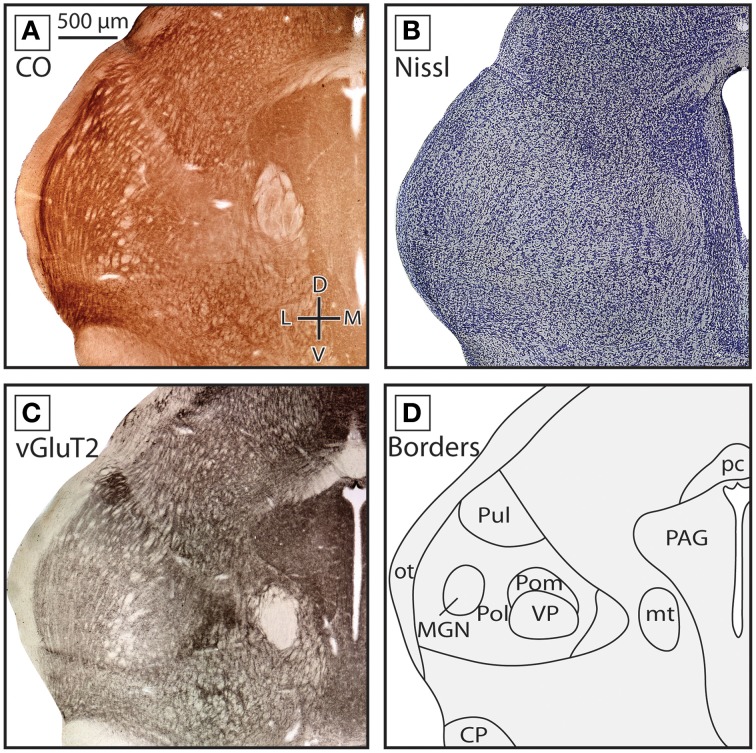
**Boundaries of nuclei in the dorsal thalamus at the caudal-most extent of the ventral posterior nucleus in the short-tailed opossum**. All three stains used are the same in Figures 3, 4, along with an illustration of nuclear boundaries. In these sections VP, Pol and MG can be identified. Pol is lateral to VP and extends outward, surrounding the emerging MGN. Conventions as in previous two figures.

**Figure 6:**
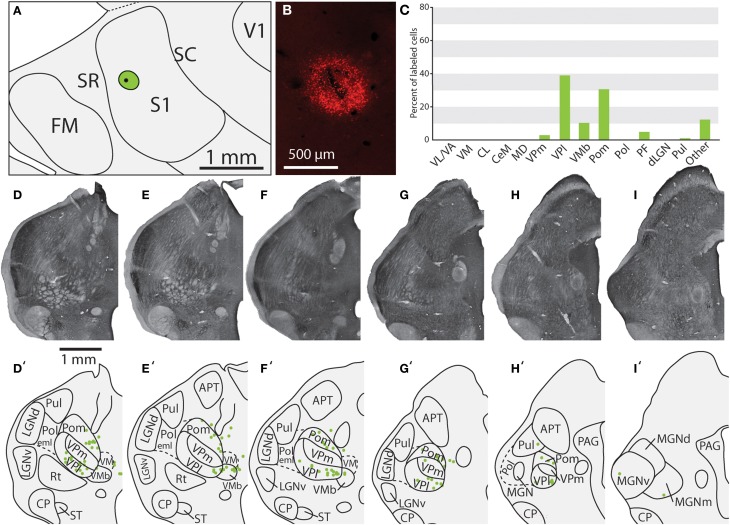
**Projections to the S1 body representation from in case 13-114**. **(A)** Reconstruction of the flattened cortical hemisphere, showing the core of the injection site of FR (black dot) surrounded by the halo (green circle) in the body representation of S1. **(B)** Fluorescent image of the FR injection site and surrounding halo. **(C)** Percent of labeled neurons originating from various thalamic nuclei (**D–I**) Rostral to caudal progression of CO stained tissue; corresponding thalamic borders and labeled neurons are shown below (**D'–I'**). In this case, the majority of label was found within VPl, VMb, and Pom. All conventions as in previous figures.

In the caudal most portions of the thalamus, Pol could be identified. In the rostral sections where it was identified, Pol was mostly intermingled with eml (see Figures 4A–C, 5A–C, 6D–G). While the boundaries of Pol were at times difficult to identify with the stains used in the present studies, in favorable sections it could be identified as a lightly staining nucleus lateral to VP but medial to LGN. In the more rostral sections Pol is bordered ventrally by Rt. Finally, the MGN began to emerge just caudal and lateral to VP. In CO and vGluT2 stained tissue, fiber bundles running perpendicular to the tissue could be seen separating MGN from VP (Figures 5A–C, 6H). As the MGN begins to emerge, the region surrounding and between these nuclei was identified as the caudal extension of Pol described above (Figures 5A–C, 6H). In more caudal sections of the thalamus MGN became larger and was located further laterally, and could be further subdivided into different divisions, including the darkly stained MGNv as well as the more lightly staining MGNd and MGNm (Figure 6I).

### Thalamic connections of the primary somatosensory area (S1)

**Figure 7:**
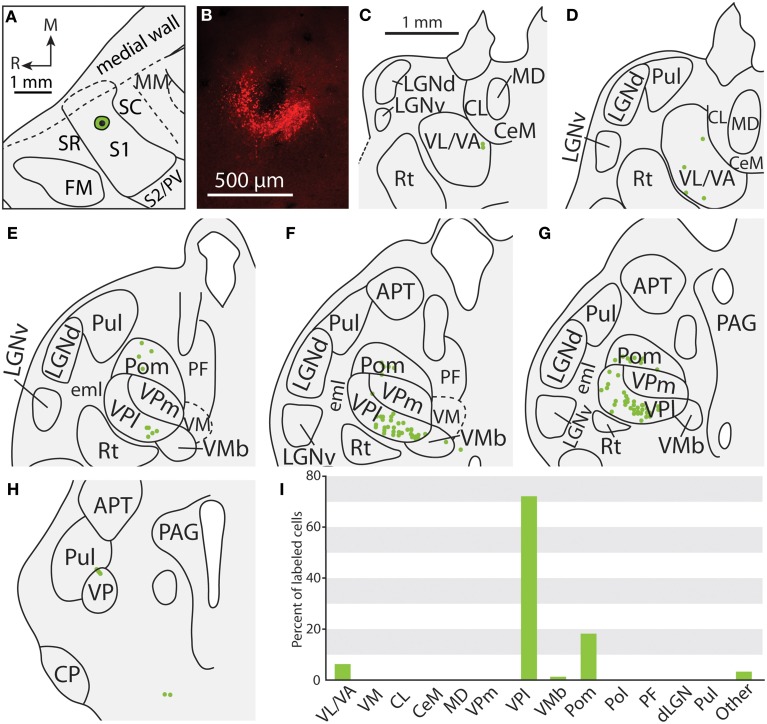
**Projections to the S1 body representation in case 12-13. (A)** Reconstruction of the FR injection into the body representation of S1. **(B)** Fluorescent image of the FR injection site and surrounding halo. **(C, D)** Distribution of labeled neurons were observed in similar nuclei to those shown in Figure 4, but were also located in more rostral portions of the thalamus in VL/VA. (**E–H**) Labeled neurons throughout VPl and Pom have a similar distribution that was illustrated in the previous figure. (**I**) Percent of labeled neurons originating from various thalamic nuclei. All conventions as in previous figures.

**Figure 8:**
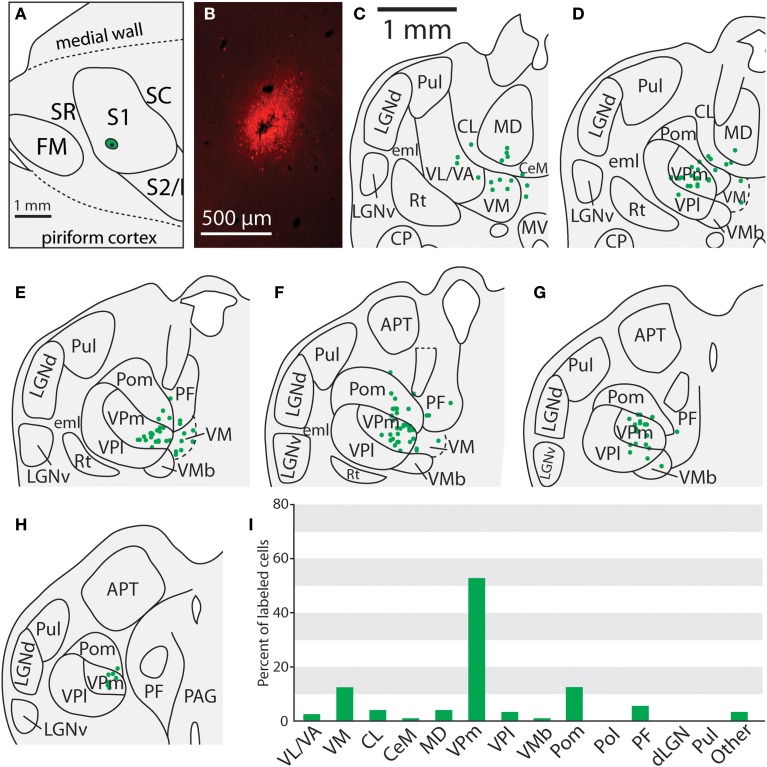
**Projections to the face representation in case 13-126. (A)** The location of an FR injection site in the lateral portion of S1 in the representation of the face. **(B)** Fluorescent image of the FR injection site and surrounding halo. (**C–H**) Progression of the thalamic sections with labeled neurons, showing labeled neurons largely restricted to VPm, Pom and VM. **(H)** Percent of labeled thalamic neurons in this experiment. **(I)** Percent of labeled neurons originating from various thalamic nuclei. Conventions as in previous figures.

**Figure 9:**
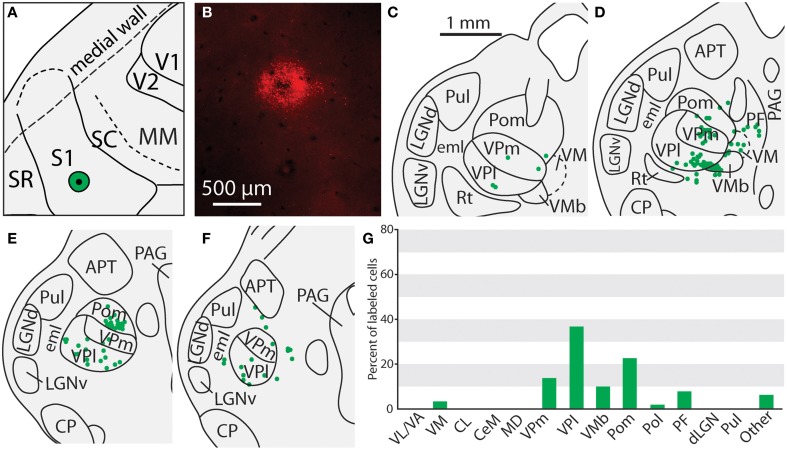
**Thalamic projections to the border of the face-body representation in S1 in case 12-18. (A)** Location of the FR injection site. **(B)** Fluorescent image of the FR injection site and surrounding halo. **(C–F)** Labeled neurons can be seen throughout the medial and lateral divisions of VP, along with labeled cells in Pom and VMb. **(G)** Percent of labeled thalamic neurons projecting to S1. Conventions as in previous figures.

Of the six cases in which injections were made in S1, two of the injections were restricted to the body representation of S1 (Figures 6, 7), two were restricted to the face representation of S1 (Figure 8; case 09-18, not shown), and two were placed across the S1face/S1body representations (Figure 9; case 13-73, not shown). In all 6 cases, the majority of projections originated from VP (mean = 57.5%, see Table 3 for the percentages from individual cases), with the percentage of labeled cells that originated from VPm and VPl varying dramatically depending on the location of the cortical injection within S1. In one case, the injection was placed in the medial portion of S1 (in the expected representation of the forepaw, case 13-114, Figure 6A), resulting in the vast majority of labeled cells residing in VPl (Figures 6D–I). Conversely, a cortical injection placed in the lateral portion of S1 (Figure 8A, confirmed with electrophysiological recording to be restricted to the vibrissae representation of the face) resulted in the vast majority of labeled cells located in VPm (Figures 8D–H). In one case, two injections were placed such that one was restricted to S1 body representation and another was restricted to S1 face representation (extending rostrally into SR, Figure 2A). In this case, VP neurons projecting to S1 face representation (dark green squares) are restricted to VPm while neurons projecting to S1 body representation (light green circles) are almost entirely restricted to VPl (Figure 2B). In nearly all cases in which S1 was injected, labeled cells were not spatially restricted to a particular portion of VPl or VPm, but were found throughout each of these subdivisions suggesting a rather loose topographic organization of these nuclear subdivisions (e.g., Figures 2B, 7G).

**Table 3:** **Percentage of labeled neurons observed in different thalamic nuclei out of total labeled neurons in the thalamus**.

**Case**	**Area injected**	**VA/VL**	**VM**	**CL**	**CeM**	**MD**	**VPm**	**VPl**	**VP**	**VMb**	**Pom**	**Pol**	**PF**	**LGNd**	**Pul**	**Other**
12-13	S1 body	6.0	0.0	0.0	0.0	0.0	0.0	72.0	72.0	1.0	18.0	0.0	0.0	0.0	0.0	3.0
13-114	S1 body	0.0	0.0	0.0	0.0	0.0	2.8	38.9	41.7	10.2	30.6	0.0	4.6	0.0	0.9	12.0
13-126	S1 face	2.3	12.2	3.8	0.8	3.8	52.7	3.1	55.7	0.8	12.2	0.0	5.3	0.0	0.0	3.1
09-18	S1 face	0.0	2.8	0.0	0.0	0.0	43.4	26.4	69.8	10.4	11.3	1.9	2.8	0.0	0.0	0.9
12-18	S1	0.0	3.0	0.0	0.0	0.0	13.4	36.6	50.0	9.7	22.4	1.5	7.5	0.0	0.0	6.0
13-73	S1	4.0	11.4	4.0	1.5	0.0	23.8	32.2	55.9	4.0	16.8	0.0	0.0	0.0	0.0	2.5
Average		2.0	4.9	1.3	0.4	0.6	22.7	34.9	57.5	6.0	18.6	0.6	3.4	0.0	0.2	4.6
SEM		1.0	2.2	0.8	0.3	0.6	8.8	9.1	4.7	1.9	2.9	0.4	1.2	0.0	0.2	1.6
13-73	SC	10.4	7.4	2.8	0.0	1.2	9.0	24.2	33.3	0.0	16.6	17.6	0.0	0.5	4.2	6.2
09-18	SC	3.5	1.2	0.0	0.0	0.0	11.0	44.1	55.1	1.2	2.8	26.4	0.0	2.4	2.4	5.1
08-29	SC	20.7	9.5	3.8	1.1	0.2	14.0	15.5	29.5	0.7	9.6	14.4	0.5	1.3	1.8	7.0
Average		11.6	6.0	2.2	0.4	0.4	11.4	27.9	39.3	0.6	9.7	19.4	0.2	1.4	2.8	6.1
SEM		5.0	2.5	1.1	0.4	0.4	1.5	8.5	8.0	0.3	4.0	3.6	0.2	0.5	0.7	0.6
08-80	MM	12.7	0.9	0.5	0.0	1.4	9.9	16.9	26.8	0.5	8.5	27.7	0.0	6.6	6.6	8.0
08-80	SR	8.9	20.6	1.1	1.2	16.1	7.4	2.9	10.3	8.4	20.1	0.0	5.5	0.0	0.1	7.7

In addition to projections from VP, all injections in S1 resulted in strong projections from Pom (mean = 18.6%) and moderate projections from VM and VMb (mean = 4.9 and 6.0% respectively). For both of these nuclei we observed a difference in the percentage of labeled neurons depending on the placement of the injection within S1. Injections restricted to the body representation in S1 did not have projections from VM, while all injections into the face representation of S1 had moderate to strong projections from VM (range = 2.8–12.2%). Projections from VMb to S1 were not present in every case, but did not appear to differ systematically for body versus face representations in S1 (see Table 3). However, the two injections with strong projections from VMb are located rostrally within S1, closer to the expected location of the representation of oral structures (Figure 6A; see Figure 1 for location of oral representation). Moderate to weak projections were also observed from PF and VA/VL, while all other nuclei in which labeled cells were found had weak and inconsistent projections to S1 (e.g., CL, CeM, see Table 3).

### Thalamic connections of the caudal somatosensory area (SC)

**Figure 10:**
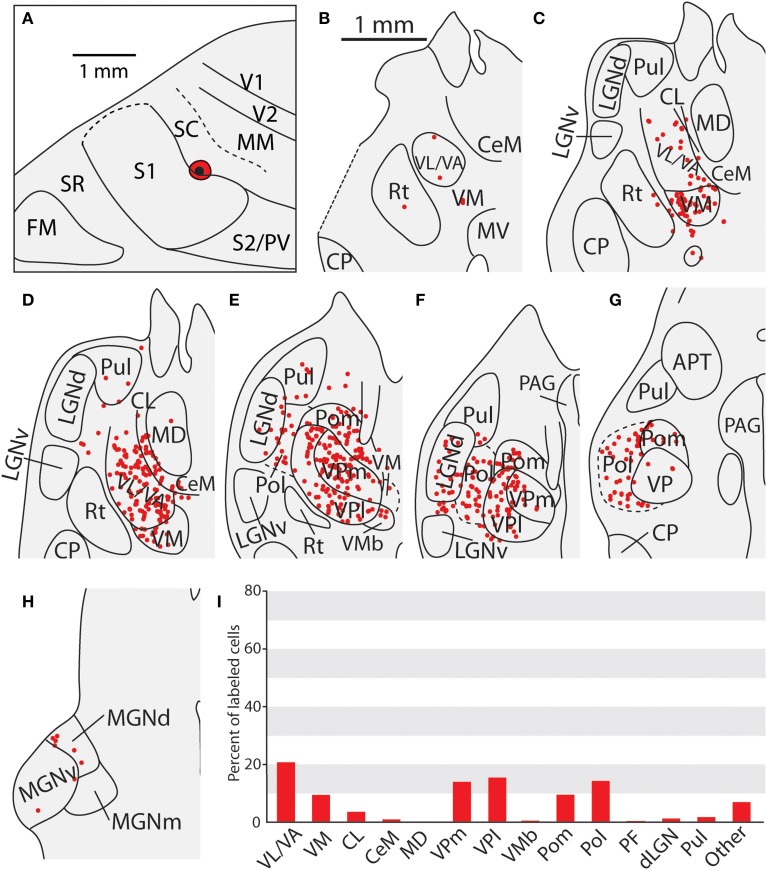
**Thalamic projections to area SC in case 08-29. (A)** An injection of FE into SC which extends slightly into the caudal-most portion of S1. **(B–D)** Densely packed labeled cells can be seen in VL/VA and VM, **(D–G)** as well as throughout VPm and VPl. Large numbers of labeled cells can also be seen throughout Pol, sometimes extending laterally into LGNd and Pul. **(H)** A small number of cells were seen projecting from the medial geniculate complex. **(I)** Percent of labeled thalamic neurons projecting to SC. Conventions as in previous figures.

**Figure 11:**
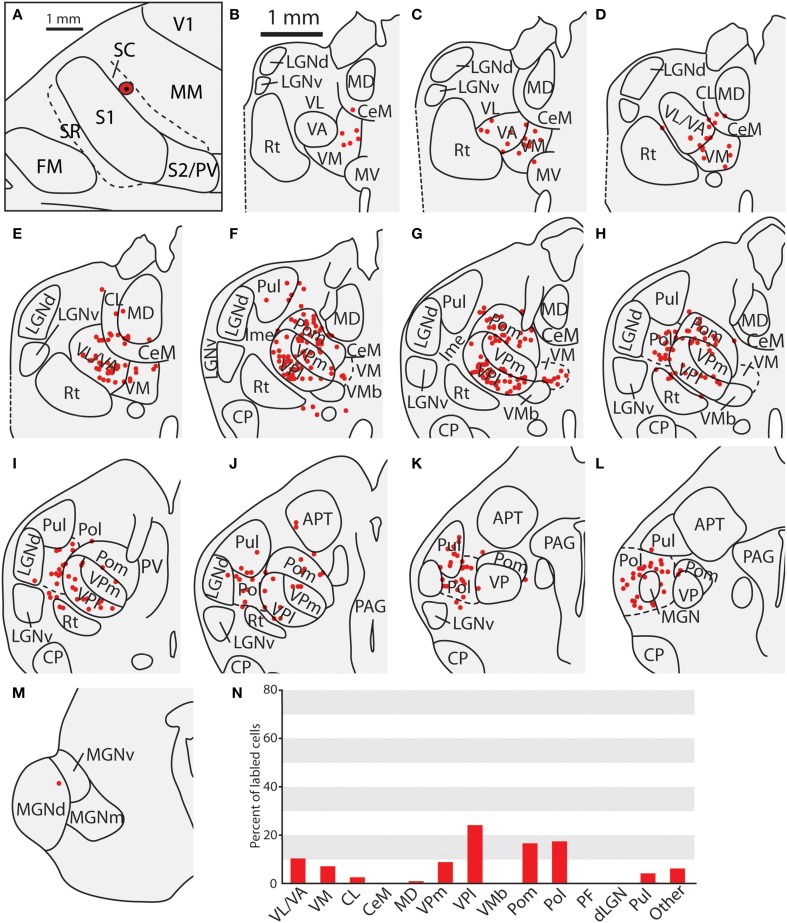
**Thalamic projections to area SC in case 13-73. (A)** Location of cortical injection sight of FR into SC. **(B–E)** Labeled cells can be seen throughout VL/VA and VM. **(F–J)** Additional numerous labeled cells can be seen throughout VPl and VPm, as well as Pom. **(K,L)** As with Figure 8, labeled cells can be seen throughout Pol, sometimes extending laterally into LGNd and Pul, along with labeled cells at the caudal-most extent Pol as the Medial Geniculate complex begins to emerge. **(M)** A single cell was observed in the medial geniculate complex. **(N)** Percent of labeled thalamic neurons projecting to SC. Conventions as in previous figures.

The three cases in which injections were restricted to SC had very consistent patterns of thalamic connectivity (Figures 10, 11, case 08-29, not shown). As with S1, the strongest projections originated from VP (mean = 39.3%), however SC also received strong projections from Pol (M = 19.4%), VA/VL (mean = 11.6%), and Pom (mean = 9.7%). These strong projections from VL/VA were not spatially restricted, and were located throughout these nuclei (Figures 10C,D). Additionally, labeled neurons in Pol were observed throughout the nucleus (Figures 10E–G, 11H–L). Moderate projections to SC also originated from VM (mean = 6.0%). Notably, weak projections from LGNd and Pul (two visual nuclei) were also present in all three cases (mean = 1.4 and 2.8% respectively). When comparing the thalamocortical projection profiles of S1 and SC, the most notable differences are the density of projections of VP, Pol and VA/VL (Table 3). S1 had more dense projections from VP while SC had more dense projections from Pol and VA/VL. Further, SC had weak but consistent projections from visual nuclei of the thalamus which were not observed for S1 projections.

### Thalamic connections of the multimodal region (MM) and the rostral somatosensory areas (SR)

**Figure 12:**
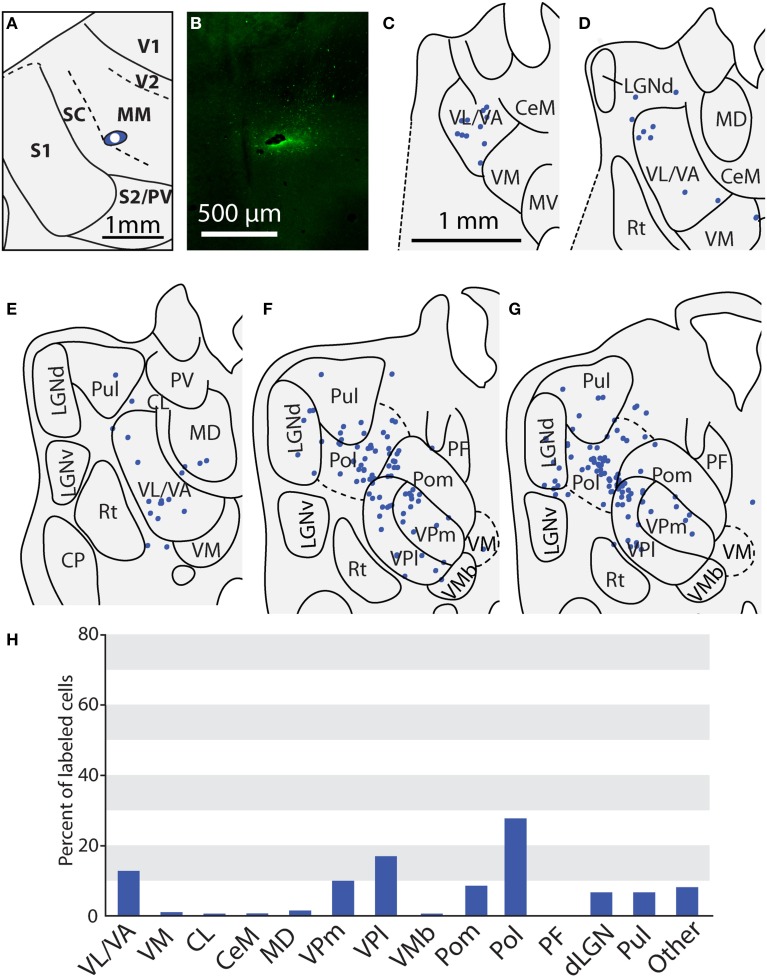
**Thalamic projections to multimodal region (MM) in case 08-80. (A)** Injection site of FE in MM. **(B)** Fluorescent image of the FE injection site and surrounding halo. **(C–G)** Labeled neurons can be seen projecting to MM from large portions of VL/VA as well as from VPl and VPm. Additionally, densely packed label neurons are observed in Pol; moderate numbers of labeled neurons in LGNd and Pul. **(H)** Percent of labeled thalamic neurons projecting from various thalamic nuclei to MM. Conventions as in previous figures.

**Figure 13:**
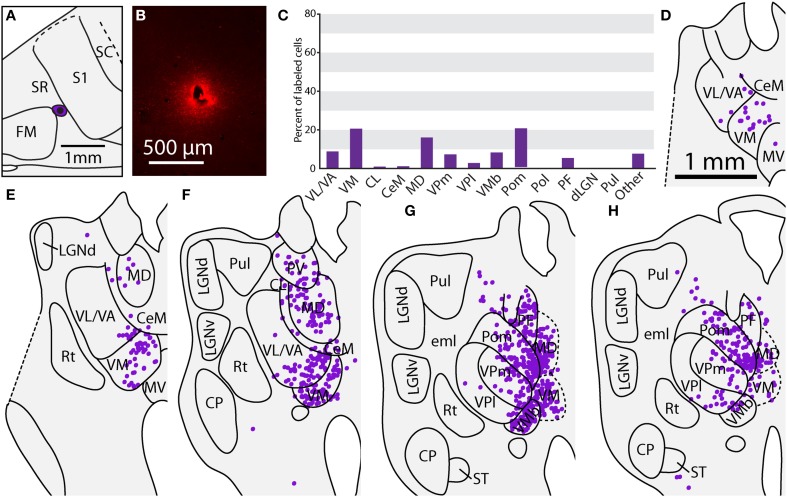
**Thalamic projections to SR in case 08-80. (A)** Reconstruction of the injection of FR in SR. **(B)** Fluorescent image of the FR injection site and surrounding halo. **(C)** Percent of labeled thalamic neurons projecting to SR. **(D–H)** Unlike previous injections, the majority of label cells were in the midline nuclei, including PV, MD, and PF. Conventions as in previous figures.

One injection was entirely restricted to MM (Figure 12A). In this case, MM received strong projections from VP and Pol (26.8 and 27.7% respectively, see Figures 10F,G). Additionally, MM also received significant projections from VL/VA (12.7%) and moderate projections from both LGNd and the pulvinar (6.6% for both nuclei). Thus, MM is distinguished from SC by its relatively dense projections from LGNd and the pulvinar, giving MM a more multimodal connectional profile compared to the primarily somatosensory SC, Additionally, we had one injection entirely restricted to SR (Figure 13). Unlike other cortical parietal areas described thus far, SR received substantially more projections from both VM (20.6%) and Pom (20.1%) than from VP (10.3%, see Figures 13D–H). SR also received very dense projections from several midline structures that do not consistently project to S1, SC, or MM. For example, SR received strong projections from MD (16.1%), while VA/VL (8.9%), VMb (8.4%), and PF (5.5%) all have moderate projections to SR. Weak projections were also observed from CL and CeM (1.1 and 1.2%, respectively).

**Figure 14:**
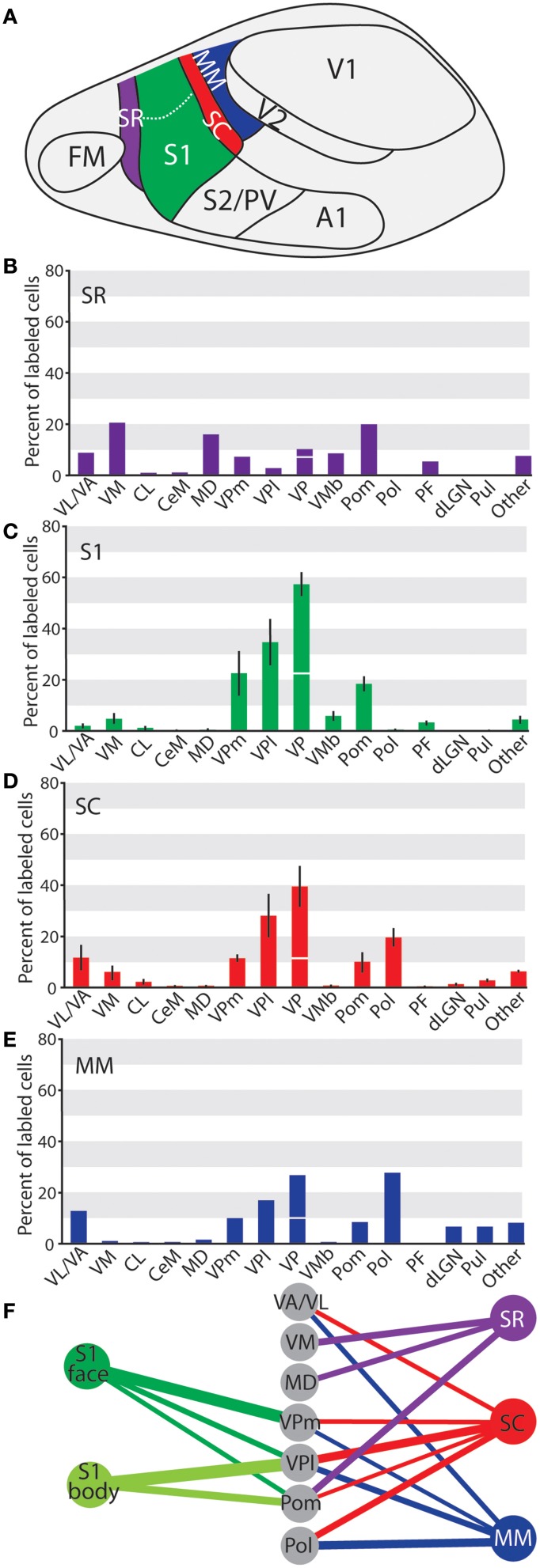
**Summary of projections across cortical areas investigated in these studies. (A)** Illustration of cortical areas, using the same colors as the graphs and diagrams below. Percent of labeled thalamic neurons projecting to **(B) SR, **(C)** S1, **(D)** SC, and **(E)** MM. Data are mean ± SEM when applicable. Axes are the same throughout A-D to aid in comparison. Projections to VPm and VPl are summed above VP, with the projections from VPm under the horizontal line and the projections from VPl above the line. **(F)** Summary of strong projections from the 5 cortical areas discussed throughout the paper. All projections shown make up > 10% of the total thalamic projections to that area**.

Taken together, these data demonstrate that S1, SC, and MM received the strongest projections from VP; however the strength of those projections decreased from S1 to SC and MM (see Figure 14, Table 3). Conversely, MM had stronger projections from visual structures (LGNd and Pul) while SC has only sparse connections from these nuclei and S1 had no projections from these nuclei. Mimicking the pattern of VP connections, the strength of projections from Pom was weaker as injections moved farther caudal in cortex (e.g., from S1 to SC to MM). Strong projections from Pol were only found in areas caudal to S1 (Figure 14F). Located immediately rostral to S1, SR showed a pattern of projections from thalamic nuclei that was notably different from the other three areas described. Strong projections to SR were seen from midline structures including VM and MD, which only weakly or inconsistently projected to the other cortical areas examined (Figures 14B,F).

## Discussion

The present investigation is part of a broader objective of our laboratory to appreciate the cortical organization and connectivity of early mammals, and how this basic plan was modified in different lineages to produce the remarkable variability in behavior observed in extant species. Comparative studies in general allow us to infer aspects of organization of our early ancestors; however, some species can provide a more accurate representation of early mammals based on their phylogenetic status, lifestyle, as well as brain and body morphology (Kemp, 2005; Kaas, 2011; Rowe et al., 2011). One such species is the marsupial *Monodelphis domestica*, or the short-tailed opossum. Previously, we examined the corticocortical connections of several somatosensory areas in *Monodelphis* and found evidence for three somatosensory fields (SR, S1, and SC) as well as a multimodal region (MM; Dooley et al., 2013). In the present experiments we investigated the thalamocortical projections of these cortical fields and demonstrate that each cortical field has a unique pattern of thalamocortical connections, supporting our parcellation of cortex. Additionally, we demonstrate different thalamic projections to the S1 body representation and the S1 face representation, which we interpret in the context of sensorimotor integration for specializations of the face versus the body in *Monodelphis domestica*. Finally, thalamocortical connections to all cortical areas examined in the present study are compared to projections in other marsupials and in other mammals to better appreciate common patterns of this thalamocortical network as well as derivations.

### Thalamic projections to S1 in marsupials

Early studies in marsupials such as the Virginia opossum demonstrated that unlike placental mammals, marsupials do not appear to have a separate cortical motor area. Rather, they possess what has been called a “somatosensory-motor amalgam” (Lende, 1963; Killackey and Ebner, 1973). The somatosensory-motor amalgam refers to the complete overlap of the Virginia opossum's topographic representation of cutaneous receptors of the body and face in the primary somatosensory area (S1) with motor maps of the body in the primary motor area (M1). Given that marsupials represent a very early mammalian radiation whose ancestors diverged over 150 million years ago (Nilsson et al., 2010), one current hypothesis is that their sensory motor amalgam represents a primitive form of mammalian neocortex, which ultimately segregated into separate sensory and motor areas in placental mammals (Kaas, 2004). While our understanding of the relationship between sensory and motor cortex has undergone enormous transformations in the last decade (Hatsopoulos and Suminski, 2011; Kaas et al., 2012), the initial observation of a complete lack of a separate motor cortex in marsupials has been validated in other marsupials including short-tailed opossums and the big-eared opossums (Magalhaes-Castro and Saraiva, 1971; Frost et al., 2000). Because it is likely that this type of organization does indeed represent a more primitive form of cortical organization, determining the connectivity, particularly with somatosensory and motor subcortical structures, is a critical step in understanding how the cortical motor system in placental mammals ultimately came to generate sophisticated motor control necessary for complex, goal-directed movements.

There are only a few studies of thalamocortical connections in marsupials. The thalamic projections to S1 in the Virginia opossum are from the VP as well as from VL/VA, nuclei associated with the motor system (Killackey and Ebner, 1973; Donoghue and Ebner, 1981b; Beck et al., 1996). Projections from VPl and VPm project to the body and face representations of S1, respectively, and a previous study in the big-eared opossum has described even finer somatotopy within VP (Sousa et al., 1971). While the current investigation did not examine somatotopy at this level of specificity, our results are consistent with this previous report. Additionally, strong projections to S1 have also been reported from the medial division of the posterior nucleus, Pom (Killackey and Ebner, 1973; Donoghue and Ebner, 1981b). In two Australian marsupials, brush-tailed possums and Northern quolls, cortical projections of VL and VP were overlapping, such that a notably large portion of parietal cortex (presumed to be S1) received projections from both nuclei (Haight and Neylon, 1978a,b, 1981a,b). Notably, for projections from both VL and VP, thalamic projections corresponding to distal body parts (e.g., digits of the paws) projected to more rostral portions of cortex, and thalamic projections corresponding to proximate body parts (e.g., the trunk) projected to more caudal portions of cortex. In contrast, in placental mammals the topography of projections to cortex from VL are mirrored relative to those of VP, with distal body parts (e.g., digits of the paws) projecting to relatively caudal portions of cortex compared to proximate body parts. Unfortunately, neither electrophysiological nor histological techniques were used to confirm the placement of injections into parietal cortex in these Australian marsupials, making thalamocortical projections from VL and VP to S1 difficult to compare across species.

These results described in other marsupials are consistent with those described in the present investigation in the short-tailed opossum with respect to VP and Pom. However, short-tailed opossums differ from other marsupials studied in that they do not have significant projections from VL/VA throughout S1, with an average of only 2% of thalamic projections originating from VL/VA, and half of S1 injections resulting in no projections from this nucleus (Table 3). The lack/inconsistency of projections from VL/VA to S1 does not appear to differ based on the somatotopic origin of the injection site, a finding that is surprising considering previous results which demonstrate a difference in the motor movements elicited following intracortical microstimulation (Frost et al., 2000; see below for further discussion).

### Thalamic projections to S1 in other mammals

Studies in monotremes, an order of mammals whose ancestors radiated approximately 220 million years ago (Van Rheede et al., 2006), reveal the diversity of sensorimotor organization of early mammals. There are only three extant species of monotremes and studies of cortical connections in these species are extremely limited. Within the neocortex, the platypus and echidna have been found to have three somatosensory areas; S1, a rostral area termed R, and a second somatosensory area, which may correspond to S2 or PV. Studies also suggest the existence of a motor area rostral to R (Krubitzer et al., 1995). Thalamic projections to S1 have only been described in two studies in the echidna; one using degeneration methods (Welker and Lende, 1980) and one using retrograde tracers (Ulinski, 1984). Welker and Lende (1980) demonstrated that lesions to S1 resulted in degenerated neurons throughout VP and in a nucleus dorsal and caudal to VP (possibly Pom). Ulinski (1984) described projections exclusively from VP to S1. Neither study describes projections from VL to S1; however the delineation of thalamic nuclei in the echidna has undergone significant revisions since these studies were performed (Mikula et al., 2008), and thus these early studies may have failed to properly differentiate the VP/VL boundary. Regardless, these studies support the basic mammalian pattern of projections from VP to S1, and suggest projections from VP and VL do not overlap in cortex in the echidna.

There are numerous studies of thalamocortical projections to S1 in placental mammals, which report slight modifications on the same basic plan of thalamocortical connectivity. Because a complete review of this literature is beyond the scope of this discussion, we will focus on the pattern of thalamic projections in small-brained placental mammals, such as rodents. Studies in rats, mice, squirrels, and naked-mole rats describe thalamic projections to S1 from VP and Pom (Wise and Jones, 1977; Krubitzer and Kaas, 1987; Henry and Catania, 2006; Liao et al., 2010). More recently, it has been demonstrated in rats that projections to S1 from the thalamus are not homogeneous; instead there are parallel pathways to different cortical layers as well as to the granular and dysgranular zones within S1. VP projects to the granular zones and Pom projects to the dysgranular zones (Liao et al., 2010; Viaene et al., 2011; Ohno et al., 2012). It has been suggested that these two thalamic pathways to cortex are functionally distinct, with VP driving activity in S1 and Pom having more of a modulatory role (Hoogland et al., 1987; Liao et al., 2010; Viaene et al., 2011; Ohno et al., 2012). The present investigation confirms that as in rodents, VP and Pom project to S1, but injections in the present study were not restricted to a particular layer of cortex and there are no architectonically distinct granular and dysgranular zones in *Monodelphis*.

Weak and/or topographically restricted projections from VL and VM to S1 have also been described in some rodent species. VL projections to the forelimb and hindlimb representations in S1 have been demonstrated in rats; these representations display properties of both S1 and M1 in this species (Donoghue et al., 1979; Cicirata et al., 1986; Aldes, 1988) and other rodents (Henry and Catania, 2006; Liao et al., 2010). Sparse projections from VM to S1 are reported in diverse rodent species ranging from rats to squirrels to naked mole rats (e.g., Donoghue et al., 1979; Krubitzer and Kaas, 1987; Giannetti and Molinari, 2002; Henry and Catania, 2006), although these projections are often weak and not found in every case. In rats, VM has been shown to project primarily to rostral cortical areas, and more weakly to parietal cortex (Herkenham, 1979). Interestingly, in naked mole rats, Henry and Catania (2006) describe weak projections from VM to the S1 forepaw representation in every case, and inconsistent projections from VM to the S1 incisor representation, although a similar pattern is not noted in other studies in rodents. Thus, despite the presence of an architectonically distinct M1 in rodents, there still appears to be some sensorimotor integration of the thalamic projections to S1 within most species of rodents that have been examined.

### Somatosensory/motor integration in short-tailed opossum

As mentioned in the introduction, the short-tailed opossum is unique among mammals investigated in that it does not have a motor representation of its entire body; only the head has a cortical motor representation (Frost et al., 2000). These results inspired our hypothesis that the body and face representations in S1 would have different patterns of thalamic projections, specifically in the case of projections from nuclei associated with the motor system such as VL/VA. As noted above, however, VL/VA only showed weak and inconsistent projections to S1, and these weak projections did not differ between S1 face and S1 body (Figures 14C,F). This finding differs from the evidence of a complete somatosensory motor amalgam previously described for the Virginia opossum, with extensive projections form VL/VA and VP throughout S1 (Killackey and Ebner, 1973). One explanation for these differences between marsupials is that connections between VL/VA and S1 have been lost in short-tailed opossums; another possibility is that the thalamocortical projection patterns in *Monodelphis* actually represent the ancestral mammalian plan. However, because both thalamic projections and functional organization have been assessed in only two opossum species we cannot distinguish between these two alternative hypotheses.

The largest difference we found was that the sensory-motor face representations received moderate projections from VM while the S1 body representation did not receive any projections from VM (Table 3, Figure 14C). In other species, VM receives input from the cerebellum and projects to numerous (particularly rostral) cortical areas as well as other subcortical structures that are part of the motor system (Donoghue et al., 1979; Herkenham, 1979; Kuramoto et al., 2013). In addition to the differential thalamic projections to the face versus the body representation in S1, we also demonstrate that the face representation receives input from both VPm and VPl while the body representation receives input only from VPl (Table 3). In our previous investigation of corticocortical connections, we observed that intrinsic connections of S1 are generally heterotropic, and thus not restricted to similar somatic representations (Dooley et al., 2013). Thus, the S1 face representation receives projections from thalamic nuclei associated with motor processing (VM) as well thalamic input from both the face and body somatosensory representations. This suggests that motor control of portions of the snout and face as well as inputs processed within the face representation of S1 together form specialized differentiated, highly integrated system that differs from the rest of the body.

When considering why the S1 face representation is so highly integrated with different cortical areas and thalamic nuclei, it is important to consider the peripheral prominence of the vibrissae and the cortical magnification of the face in light of its ethological significance. Studies of whisking behavior in short-tailed opossums have found that they do display active vibrissal exploratory behavior (Mitchinson et al., 2011; Grant et al., 2013). This suggests that prominent sensory face hairs may have been a shared feature of the common ancestor of placental and marsupial mammals, and thus may be one of the earliest and most pronounced forms of sensory reception. Ultimately, the representation of vibrissae within the cortex may be an example of a significant, and perhaps ubiquitous, site of sensorimotor integration. As mammalian species radiated, different morphological structures and associated sensory arrays, such as the hands and eyes in primates, have undergone specialization and an accompanying cortical expansion of motor and posterior parietal cortex (see Cooke et al., 2014). However, the organization and connectivity of the opossum vibrissae system suggests that strong sensorimotor integration of ethologically significant body parts has long been a general feature of the mammalian neocortex.

### Thalamic projections to SR, SC, and MM in other species

Among marsupials, SR and SC have been identified based on receptive field characteristics and stimulus preference in the Virginia opossum (Beck et al., 1996), the brush-tailed opossum (Elston and Manger, 1999), the northern quoll, the striped possum (Huffman et al., 1999b), and the big-eared opossum (Anomal et al., 2011). Across the species investigated, neurons in SR and SC respond predominantly to stimulation of deep receptors in muscles and joints, show a coarse topographical organization, and have larger receptive fields compared to neurons in S1. In most of these studies, these functionally defined fields have been directly related to cortical architecture (see above) and in some species to cortical connections. Corticocortical connections of both SR and SC have been reported in the Virginia opossum (Beck et al., 1996), the brush-tailed possum (Elston and Manger, 1999), the short-tailed opossum (Dooley et al., 2013), and for SC, the big-eared opossum (Anomal et al., 2011). In all of these studies, the pattern of projections provided further evidence that SR and SC are distinct cortical fields and part of a somatosensory network. It has been proposed that SR is homologous to 3a or dysgranular cortex in rodents and SC is homologous to posterior parietal cortex in rats (for review, see Karlen and Krubitzer, 2007; Krubitzer et al., 2011). Our studies of corticocortical connections as well as previous electrophysiological recording studies demonstrate an additional region of cortex just caudal to SC in which neurons respond to more than one modality of stimulation (Huffman et al., 1999a; Kahn and Krubitzer, 2002), and which receives input from visual and somatosensory cortex (Dooley et al., 2013). We term this field MM or the multimodal region.

Despite consistent evidence for both SR and SC in all marsupials studied, only one study in marsupials examined the thalamocortical connections of these fields (Virginia opossum; Donoghue and Ebner, 1981b). In this early study, several injections were placed immediately rostral and caudal to S1. The rostral injections sites were in an area termed post-orbital cortex (likely equivalent to SR in the present investigation) and projections to this area were from several midline and intralaminar nuclei including CL, PF, MD, VL, and Pom (identified as CIN), with sparse projections from VP (see Figure 9 in Donoghue and Ebner, 1981b). These projections are consistent with the patterns of projections we observed, only with a smaller contribution from CL and more projections from Pom and VMb. Additionally, while in the present investigation only one injection was found to be entirely restricted to SR, numerous injections spanned SR and S1 (e.g., the light green injection in Figure 2A), showing a similar pattern of thalamic projections, just with a greater percentage of neurons originating from VP (Figure 14B).

As mentioned above, we have proposed that SR may be homologous to dysgranular cortex (3a) in rodents. In both rats and squirrels, dysgranular cortex receives strong projections from Pom, along with moderate projections from VP, VL/VA, and CL, as well as projections from VM (Koralek et al., 1988; Gould et al., 1989). Notably, dysgranular cortex does not receive projections from midline nuclei MD and PF. However, apart from this exception projections to dysgranular cortex/3a are similar to those to SR in short-tailed and the Virginia opossums.

Donoghue and Ebner (1981b) did not delineate cortical areas caudal to S1, instead referring to the region caudal to S1 and rostral to peristriate cortex (V2) as posterior parietal cortex, or PP. Their injection, however, appears to be in a location similar to MM in the present study rather than to SC, since it borders the rostral edge of peristriate cortex (see Figure 8 in Donoghue and Ebner, 1981b). They report projections from VL/VA, Pol (termed Po), and weak projections from intralaminar nuclei. Notably, they do not see any labeled neurons in VP (termed VB) following any injections into PP. In the present investigation, both SC and MM receive strong projections from VL/VA and Pol, as was found in the Virginia opossum, however projections differ in two important ways (Figures 14D–F). First, there are still strong projections from VP to both SC and MM, although not as strong as was found for S1. Second, both of these areas (although especially MM) display consistent connectivity with visual thalamic nuclei LGNd and the pulvinar. Thus, the general trend of projections from the thalamus to SC and MM in the short-tailed opossum is consistent with projections described in PP of Virginia opossum. However, the short-tailed opossum shows projections from primary sensory relay nuclei from both the visual and somatosensory systems not found in Virginia opossums, further suggesting that these thalamic primary sensory nuclei display more exuberance in their projections in short-tailed opossums compared to other species.

This exuberance of the projections of these primary sensory nuclei in short-tailed opossums compared to other marsupial and mammalian species builds upon previous work in cortex that suggests that brain circuits become more segregated as the brain increases in size (Ringo, 1991). Increased long range connections have been documented in small-brained rodents (Campi et al., 2010; Henschke et al., 2014) as well as primates (Palmer and Rosa, 2006). Thus, it is possible that the observed exuberance of thalamocortical connectivity in the short-tailed opossum is due to the small size of their brain. Whether this thalamocortical exuberance in the small-brained short-tailed opossum is unique to the species, to small-brained marsupials, or a shared property across other groups of mammals requires a larger comparative analysis.

The location and thalamocortical connectivity of *Monodelphis* MM and SC (PP in Virginia opossums) suggest that they are homologous to some portion of posterior parietal cortex described in eutherian mammals. Posterior parietal cortex is a region of the mammalian neocortex which has undergone massive expansion in primates, and thus has been of interest to numerous researchers interested in the organization of the human brain (see Cooke et al., 2014 for review). Despite this, comparing SC and MM in the present investigation to posterior parietal areas in other species is challenging, as very little is known about the functional role of these areas in opossums and other small-brained mammals such as rodents (see Krubitzer et al., 2011). There is, however, an area with similar cortical architecture and connections that has been identified in rats (termed posterior parietal cortex, or PPC; Reep et al., 1994) and squirrels (termed parietal medial area, or Pm; Slutsky et al., 2000). Among small-brained animals, PPC in rats has been the most studied, where, numerous behavioral studies have implicated PPC as part of a larger thalamo-cortical-basal ganglia network that plays an important role in multimodal spatial attention (Reep and Corwin, 2009). Thalamic projections to PPC include the lateral dorsal nucleus and the lateral posterior nucleus (pulvinar), as well as projections from VL and Po (Giannetti and Molinari, 2002). Notably, despite close proximity to both S1 and peristriate cortex in rat, PPC does not receive projections from VP or the LGNd. Like PPC in rats, MM in *Monodelphis* has strong projections from Pol and VL/VA, along with moderate projections with pulvinar (lateral posterior nucleus in rat).

The finding that VL projects to posterior parietal areas has also been described in rats, cats, and monkeys, although it has been suggested that different populations of neurons within VL are projecting to motor cortex and parietal areas (Divac et al., 1977; Kasdon and Jacobson, 1978; Hendry et al., 1979; Giannetti and Molinari, 2002). Considering the finding that posterior parietal areas receive projections from VL/VA in the present investigation, we provide additional support for the idea that MM in the short-tailed opossum may be homologous to portions of posterior parietal cortex in other mammals, in part due to its connectivity to somatosensory, visual and motor nuclei of the thalamus. Further, this finding in *Monodelphis* provides further evidence that the mammalian ancestor of both marsupials and placentals possessed a cortical area similar to posterior parietal cortex, with a role integrating visual, somatosensory and motor inputs.

## Conclusion

In summary, thalamic projections to parietal cortical areas in the short-tailed opossum are similar not only to other marsupials, but other small-brained eutherian mammals. Thus, these results provide further evidence for homologies between 3a and SR as well as PP and MM in the short-tailed opossum, suggesting that these regions were present in early mammals. The projections to these regions, however, differ in short-tailed opossums in two notable ways. First, unlike other marsupials studied, there are not extensive projections from VL/VA to S1. This result highlights the need for additional comparative studies to more accurately infer the cortical organization of the common mammalian ancestor. Second, in areas such as SR and SC, as well as MM, there are strong projections from VP, along with direct projections from LGNd to MM. This highlights what appears to be a more general feature of the brain of the short-tailed opossum: Increased exuberance of the thalamic projections from primary sensory relay nuclei to non-primary cortical areas. This finding suggests that our small-brained mammalian ancestors may have also possessed increased *thalamocortical* connectivity, similar to the increased *cortical* connectivity found in many extant small-brained mammals. However, we underscore the need for additional comparative studies of mammals in order to determine whether this feature has independently evolved in small brained mammals, or is a retained ancestral trait.

## Author contributions

All authors had full access to the data in the study and take responsibility for the integrity of the data and the accuracy of the data analysis. Study concept and design: James C. Dooley, João G. Franca, Dylan F. Cooke, and Leah A. Krubitzer. Acquisition of data: James C. Dooley, João G. Franca, Adele M. H. Seelke, Dylan F. Cooke, and Leah A. Krubitzer. Histological processing of tissue: James C. Dooley, João G. Franca, Dylan F. Cooke, and Leah A. Krubitzer. Analysis and interpretation of data: James C. Dooley, João G. Franca, Adele M. H. Seelke, Dylan F. Cooke, and Leah A. Krubitzer. Drafting of the article: James C. Dooley and Leah A. Krubitzer. Critical revision of the article for important intellectual content: James C. Dooley, João G. Franca, Adele M. H. Seelke, Dylan F. Cooke, and Leah A. Krubitzer. Obtained funding: Leah A. Krubitzer. Study supervision: Leah A. Krubitzer.

## Funding

This project was supported by funds to Leah Krubitzer from NINDS (R21 NS071225) and NEI (R01 EY022987); funds to João Franca from CNPq—Brazil and FAPERJ—Brazil, and funds to James Dooley from NEI (T32-EY015387-05).

### Conflict of interest statement

The authors declare that the research was conducted in the absence of any commercial or financial relationships that could be construed as a potential conflict of interest.
